# Pens versus syringes to deliver insulin among elderly patients with type 2 diabetes: a randomized controlled clinical trial

**DOI:** 10.1186/s13098-021-00675-y

**Published:** 2021-06-12

**Authors:** Rafael Vaz Machry, Gustavo Fonseca Cipriani, Henrique Umpierre Pedroso, Rafaela Ramos Nunes, Thayme Luisa Souza Pires, Raquel Ferreira, Betina Vescovi, Gabriela Pereira de Moura, Ticiana Costa Rodrigues

**Affiliations:** 1 Post Graduate Program in Medical Sciences–Endocrinology, Universidade Federal Do Rio Grande Do Sul (UFRGS), Porto Alegre, Brazil; 2 Present Address: Department of Internal Medicine, Medical School, Universidade Federal de Santa Maria, Avenida Roraima 1000, Santa Maria, RS Brazil; 3 Medical School, Universidade Federal de Santa Maria, Santa Maria, RS Brazil; 4 Medical School, Universidade Federal do Rio Grande do Sul, Porto Alegre, RS Brazil; 5 Division of Endocrinology, Hospital de Clínicas de Porto Alegre, Porto Alegre, RS Brazil

**Keywords:** Type 2 Diabetes, Elderly, Pen devices, Insulin, Adherence, Glycemic control

## Abstract

**Background:**

Diabetes mellitus (DM) is a prevalent disease among elderly population. As the disease progresses, insulin may become necessary. The use of pens application seems to be more practical. However, the influence of this method on glycemic control needs to be defined in elderly people.

**Methods:**

Randomized clinical trial comparing pens and syringes for insulin application among patients with type 2 DM over 60 years old and Glycated Hemoglobin > 8.5% at baseline. The follow-up was 24 weeks, with monthly medical visits to adjust the treatment. All patients received insulin NPH and, if necessary, insulin Regular. We assessed glycemic control, adherence to treatment, hypoglycemia occurrence, need for adjustment in treatment and impact on quality of life,

**Results:**

We included 121 patients with mean age of 65.75 years. Sixty-one were randomized for pen group (PG) and 60 patients for syringe group (SG). At baseline, mean HbA1c was 10.34 ± 1.66% and 9.90 ± 1.25% (p = 0.103) in PG and SG respectively. Mean HbA1c was 8.39 ± 1.28% in PG and 8.85 ± 1.74% in SG (p = 0.101) at 24 weeks. However, there was a more significant reduction in PG (− 1.94 ± 1.93% in PG and − 1.04 ± 1.46% in SG, p < 0.05) during follow-up. We found no difference in treatment adherence rates, hypoglycemia, greater need for insulin doses or oral medication, and progression to basal-bolus insulin scheme. We also found no difference in the impact of the disease on quality of life between groups.

**Conclusion:**

Although we did not find any difference in the impact on quality of life, frequency of hypoglycemia or adherence, the PG showed a reduction in HbA1c higher in 24 weeks of follow-up.

*Clinical trial registration*: NCT02517242

## Background

Diabetes mellitus (DM) is a disease of high prevalence and population aging is a significant contributor to the growth of DM [1]. During the clinical course of the disease and over the years a reduction/failure of the pancreatic beta cells occurs and treatment with insulin becomes necessary. Regardless of age, insulin therapy may be combined with oral antihyperglycemic agents in simple or more complex forms of use [2]. However, barriers associated to this treatment with insulin may impair glycemic control. Fear of hypoglycemia, interference in daily activities, greater number of applications per day and economic issues can cause difficulty in getting adequate management of disease [3].

Among elderly patients, the type of device used appears to be relevant, those who started insulin in pens had better adherence compared to patients who used syringes [4]. However, the effect is uncertain among patients who use insulin for many years, that is, whether switching to pens could add adherence to treatment, especially in elderly patients.

The effect of the use of pens for the application of insulin in glycemic control has been investigated by several groups [5–7]. A recent meta-analysis [8] showed a small superiority in reducing Glycated Hemoglobin among patients with type 2 DM who used insulin pens compared to the use of syringes. However, these results do not appear to be clinically relevant neither for patients already on insulin nor for those starting treatment. On the other hand, the same study shows a reduction of hypoglycemic events and better adherence to treatment in favor to insulin pens.

In Brazil, the distribution of pens to apply insulin to the Public Health System is recent. In addition, the insulins available for free to type 2 DM patients are NPH and Regular. Insulin analogues are provided only for patients with type 1 DM or special situations, such as frequent hypoglycemia. Most of the studies that evaluated the effect of pens were performed with insulin analogs, in younger populations [5–8]. This is not the reality of most elderly Brazilians with type 2 diabetes.

The objective of this study was to compare the use of pens vs. syringes to apply insulin among elderly with uncontrolled type 2 diabetes treated in the public health system of southern Brazil. We evaluated the effect of the insulin application methods on glycemic control, risk of hypoglycemia, adherence to treatment and impact on quality of life.

## Methods

### Study overview

This was randomized clinical trial-not blinded-developed in two centers in the South of Brazil. We evaluated the use of pens for insulin application for glycemic control among elderly patients with T2D for 24 weeks. This study was registered in clinicaltrial.gov: NCT02517242 and approved by Research Ethics Committee of Hospital de Clínicas de Porto Alegre (Number 13-0485) and Universidade Federal de Santa Maria (Number 2.013.974). All patients agreed to participate and signed the Informed Consent Form. All participants received a copy of the document and another copy was filed with the main researcher. Funding was provided by Conselho Nacional de Pesquisa (CNPq)–Brazil and Fundo de Incentivo à Pesquisa (FIPE) at Hospital de Clínicas de Porto Alegre.

### Study population

We selected patients with T2D from 60 years old (considered elderly in Brazil). These patients should have HbA1c greater or equal to 8.5% for the last 3 months. For inclusion, subjects should be using basal insulin NPH only or basal-bolus scheme with NPH and Regular, in combination with oral antihyperglycemic (In Brazil, metformin and sulfonylureas are generally used free of charge) or already have a prescription to initiate insulin use. We excluded patients with a glomerular filtration rate less than 30 ml/min/1.73m^2^ by MDRD, who used insulin analogues, already used pens to application of insulin or who considers themselves inept to self-administer the insulin, needing caregivers. Patients using insulin pump or using a tissue glucose sensor were also not included. Outpatients were consecutively selected from those who attended at Endocrinology Division of Hospital de Clínicas de Porto Alegre and Hospital Universitário de Santa Maria. Researchers daily reviewed the electronic medical records, and invited patients who met the criteria for inclusion.

### Randomization

Patients were randomized to receive syringes or pens for insulin application throughout follow-up. Randomization was done in fixed blocks with sealed brown envelopes. Patients were randomly selected in order of entry into the study in blocks of 16 patients. All having the same probability of being included in any group.

### Study procedures

Patients in the "pen group" (PG) received pens for application of insulin of the brand Luxura®, Eli Lilly. If the subject applied basal insulin (NPH) associated with fast-acting insulin (Regular), two pens of different colors were provided. They also received 8 mm needles for the pens. In the “syringe group” (SG), we provided syringes and needles with 8 mm for insulin application. Both groups received capillary glycemia monitors, lancets and reagent tapes to measure glycemia three times a day. Vials of insulin were provided to all patients. During all visits, patients received training to use of pens or syringes, aspiration of insulin vials, technique for measuring glucose, rotation of insulin injection sites, cleaning and storage of supplies. These recommendations were given by the study monitors.

Clinical evaluations were done monthly for 24 weeks by same endocrinologist for all patients (RVM and GFC). For individuals who had not used insulin previously, NPH insulin was initiated in 12 units at bedtime on the first visit. Other patients remained with the same dosages of insulin used previously for the first month. Thereafter, the insulin dose adjustment was done at each visit. To morning glucose, there was adjustment in 4 units at the nocturnal dose of NPH insulin to achieve glucose between 70 and 130 mg/dl. If this target was not found before lunch or dinner, the NPH insulin dose should be adjusted by 8 units before breakfast or lunch, respectively. When the NPH insulin dose was greater than 40 units for application, regular insulin was started at the dose of 4 units or adjusted at 2 units for those patients already in use (basal bolus scheme). For those patients who were only using the bedtime dose of NPH insulin and remained under inadequate control throughout the day, NPH insulin dose was initiated in 12 units before breakfast. This protocol was created by the same researchers in a previous study [9].

In all evaluations, patients were questioned about the presence of hypoglycemia, severity of the episodes, symptoms presented and nocturnal episodes, and other adverse effects. The subjects underwent clinical review with blood pressure measurement (two measurements in both arms with calibrated sphygmomanometer), weight and height to calculate body mass index (BMI) in kg/m^2^. At each visit, prescribed insulin doses and other medications were reviewed. Patients returned the used insulin vials on subsequent visits. The remaining doses were counted to measure adherence to treatment. We use a syringe to empty the vials and count unit by unit returned [9]. We consider the use of at least eighty percent of prescribed dose as adequate adherence.

To evaluate quality of life and impact of the disease, we used validated questionnaires in Portuguese. All participants answered the DQOL (Diabetes Quality of Life) and BPAID (Problems Areas in Diabetes – Brazil) questionnaires on their first and last visit [10, 11].

### Laboratory evaluation

To assess diabetes control, we measured HbA1c before randomization (baseline), and at 12 and 24 weeks of follow-up. Dosages were done by HPLC ion exchange.

### Study endpoints

The first endpoint evaluated was Glycated Hemoglobin difference between groups after 12 and 24 weeks of follow-up. Therefore, the study did not have a HbA1c target. Differences in the reduction over the same period was also compared. As secondary outcomes, we evaluated the reduction in the weekly frequency of severe, asymptomatic and nocturnal hypoglycemia. We also assessed the differences in use of medications, weight gain during follow-up, insulin doses, adherence to insulin application, and disease impact on quality of life.

### Statistical analysis

The calculated sample was of 56 patients for each group (n = 112) to find a difference of at least 1% in HbA1c after 24 weeks of follow-up with a power of 90% and an alpha error of 5%. All analysis were done by intention-to-treat.

Continuous variables were tested for normality and described as mean and standard deviation (SD) and analyzed of student t-test. Variables with non-normal distribution were described as median and interquartile range (P25–75) and analyzed by Mann–Whitney test. Categorical variables were registered as number of cases (percentage) and analyzed using Chi-Square test. Generalized Estimated Equation with Bonferroni correction was used to analyze repeated measurements. Analysis were done in SPSS 18.0 (Chicago, IL).

## Results

### Study population and treatment

One hundred and sixty-one patients were consecutively invited to participate in the study, and 121 accepted. The mean age of the group was 65.75 years of age, 18.05 years of diabetes with HbA1c of 10.06% at baseline. Regarding the presence of chronic complications, 27.35% had history of ischemic heart disease. Retinopathy was described in 32.32%, nephropathy in 47.27% and neuropathy in 33.34% of the sample. Sixty-one were randomized for PG and 60 patients for SG. Only 11 participants were insulin-naive among all included patients (7 in PG and 4 in SG). Characteristics of the sample are presented in Table 1, with no difference between groups.

**Table 1:** Baseline characteristics of the study population

	Pen group	Syringe group	(p value)
Age in years (mean ± SD)	64.43 ± 12.60	67.68 ± 5.09	0.660
Male sex (%)	37.7	33.34	0.171
Race (%)			
Caucasian	60.6	70	0.083
African-descendant	6.6	13.3	
Others	32.8	16.7	
Religion (%)			
Catolics	70.4	61.7	0.161
Evangelic	19.7	20.0	
Spiritualists	6.60	5.00	
Others	3.30	13.3	
Family income*(%)			
Up to 1 minimum wage	11.4	15	0.199
1–2 minimum wage	44.3	58.3	
Over 2 minimum wages	44.3	26.7	
Education level in years (%)			
Uneducated or less than 1 year	8.2	11.7	0.753
1 to 3 years	16.4	13.2	
4 to 8 years	45.9	51.7	
9 years or more	29.5	23.4	
History of smoking (%)			
Never smoking	57.4	66.6	0.364
Current smoking	1.60	3.40	
Former	41.0	30.0	
Alcohol consumption (%)			
Never drinking	54.1	55.0	0.999
Social considers	27.9	26.7	
Alcohol abusers	1.60	1.70	
former	16.4	16.6	
Diabetic Retinopathy^a^ n and (%)			
Absent	32/50 (64.0)	35/49 (71.4)	0.536
Mild or moderative, non proliferative	9/50 (18.0)	8/49 (16.3)	
Severe, non proliferative	2/50 (4.00)	−(0.00)	
Proliferative	7/50 (14.0)	6/49 (12.3)	
Diabetic Nephropathy^b^ n and (%)			
Absent	28/51 (54.9)	30/59 (50.8)	0.088
Albuminuria increased	15/51 (29.4)	21/59 (35.6)	
Albuminuria greatly increased	6/51 (11.8)	8/59 (13.6)	
Nephrotic albuminuria	2/51 (3.90)	−(0.00)	
Presence of diabetic neuropathy^b^ n and (%)	13/49 (26.5)	21/53 (39.6)	0.413
Presence of cerebrovascular disease^c^ n and (%)	13/58 (22.4)	5/56 (8.90)	0.077
Presence of ischemic cardiopathy^d^ n and (%)	17/57 (29.9)	15/60 (25.0)	0.353
Time of diabetes in years (mean ± SD)	16.89 ± 9.64	17.83 ± 9.45	0.589
Time using insulin in years (mean ± SD)	8.89 ± 8,94	8.22 ± 8.73	0.686
Glycated hemoglobin (mean ± SD)	10.34 ± 1.66	9.90 ± 1.25	0.103
Familial history of type 2 diabetes n and (%)	47/61 (77.0)	43/60 (71.7)	0.537
Presence of hypertension n and (%)	57/61 (93.4)	53/60 (88.3)	0.464
Number of antihyperglycemic agents (mean ± SD)	2.8 ± 1.54	2.58 ± 1.32	0.409
Classes of antihyperglycemic agents (mean ± SD)	1.17 ± 0.49	1.47 ± 0.62	0.050
Time of Hypertension in years (mean ± SD)	17.07 ± 8.36	14.67 ± 8.87	0.148
Number of used drugs (mean ± SD)	7.66 ± 2.21	7.52 ± 2.38	0.721
Number of antihypertensives (mean ± SD)	2.80 ± 1.54	2.58 ± 1.32	0.409
Number of use tablet (mean ± SD)	12.24 ± 4.73	11.62 ± 4.93	0.625
Insulin dose per kg/day (mean ± SD)	0.76 ± 0.33	0.63 ± 0.39	0.056
BMI (Kg/m^2^) (mean ± SD)	31.35 ± 4.35	31.53 ± 5.03	0.830
Systolic blood pressure in mmHg (mean ± SD)	139.05 ± 15.62	133.46 ± 18.22	0.072
Diastolic blood pressure in mmHg (mean ± SD)	79.16 ± 12.37	73.72 ± 11.03	0.012

^*^minimum wage equal to $ 220.70 (reference year = august/2015)

^a^chart review

^b^chart review; we consider albuminuria > 14 mg/g of creatinine (albuminuria increased) and > 140 mg/g of creatinine (albuminuria great increased), and neuropathy to patients with description of positive monofilament test, sensorial changes or suggestive lesions

^c^history of transient ischemic attack or stroke

^d^history of unstable angina, acute myocardial infarction or diagnosis of ischemic heart disease

### Diabetes control

**Fig. 1:**
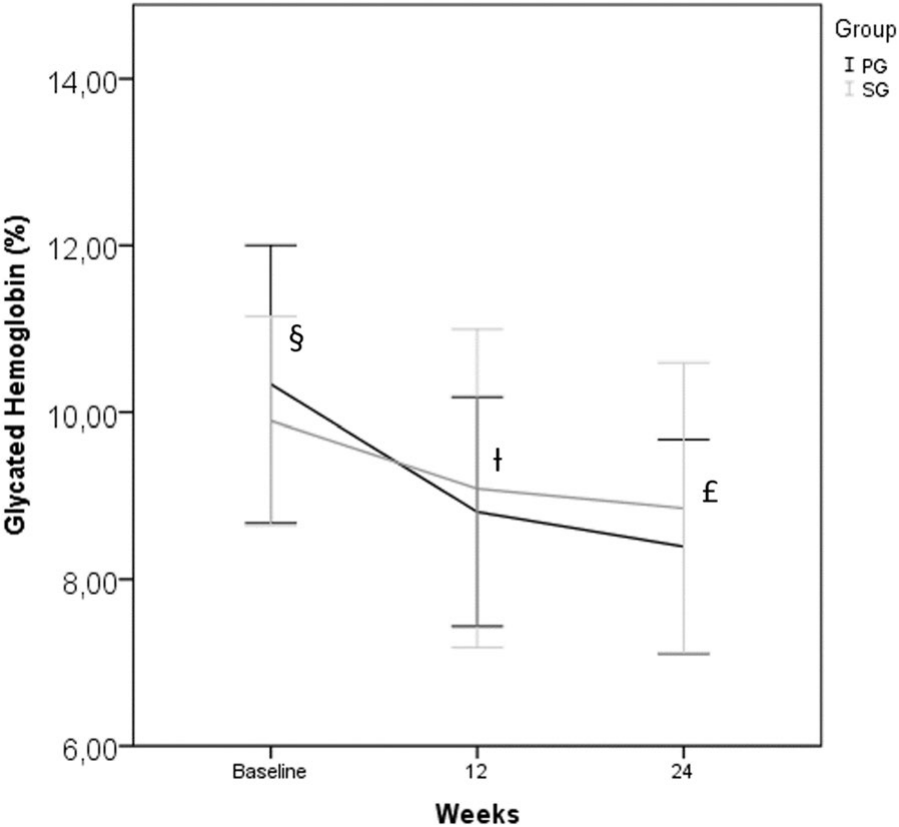
Glycated hemoglobin (HbA1c). Mean ± SD. § Compare PG with SG at baseline, p = 0.103. ƚ Compare between groups in 12 weeks, p = 0.359. Delta of HbA1c at baseline to 12 weeks: PG vs. SG, p = 0.02. £ Compare between groups in 24 weeks, p = 0.101. Delta of HbA1c at 12 to 24 weeks: PG vs. SG, p = 0.274. Delta of baseline to 24 weeks: PG vs. SG, p = 0.005

At baseline, mean HbA1c was 10.34 ± 1.66% and 9.90 ± 1.25% (p = 0.103) in PG and SG respectively. Mean HbA1c was 8.80 ± 1.37% in PG and 9.09 ± 1.91% in SG (p = 0.359) at 12 weeks, and 8.39 ± 1.28% in PG and 8.85 ± 1.74% in SG (p = 0.101) at 24 weeks. Although no significant difference was found in absolute HbA1c values between groups, the reduction was higher in PG in relation to baseline. In the first 12 weeks, there was a reduction of − 1.53 ± 1.71% in PG and -0.81 ± 1.64% in SG (p = 0.02). In 12 to 24 weeks, the reduction did not appear to be different between the groups (− 0.42 ± 0.77% in PG and − 0.23 ± 1.02% in SG; p = 0.274). Regarding the entirety of the follow-up period, there was a more significant reduction in PG (− 1.94 ± 1.93% in PG and − 1.04 ± 1.46% in SG, p < 0.05). There was a 18.76% and 10.5% (p < 0.05) reduction of the initial HbA1c in the total follow-up period in PG and SG, respectively see Fig. 1.

### Clinical outcomes

**Fig. 2:**
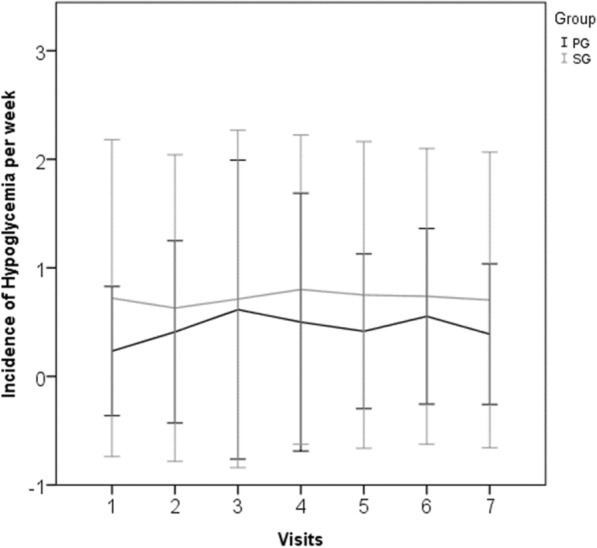
Incidente of hypoglycemia per week

Despite a trend towards greater number of hypoglycemia cases in SG, no difference was found in relation to PG. The weekly incidence of hypoglycemia was less than 1 episode per week in both groups and did not change throughout the study compared to baseline. These results are in Fig. 2. There was no difference between groups in the occurrence of severe, asymptomatic, or nocturnal hypoglycemia during the follow up.

We compared the adherence to the prescribed insulin dose. There was no statistically significant difference between groups. On the other hand, SG reached an adequate adherence average (use of more than 80% of the prescribed dose) only after the fifth visit. PG presented mean greater than 80% from the third visit.

**Fig. 3:**
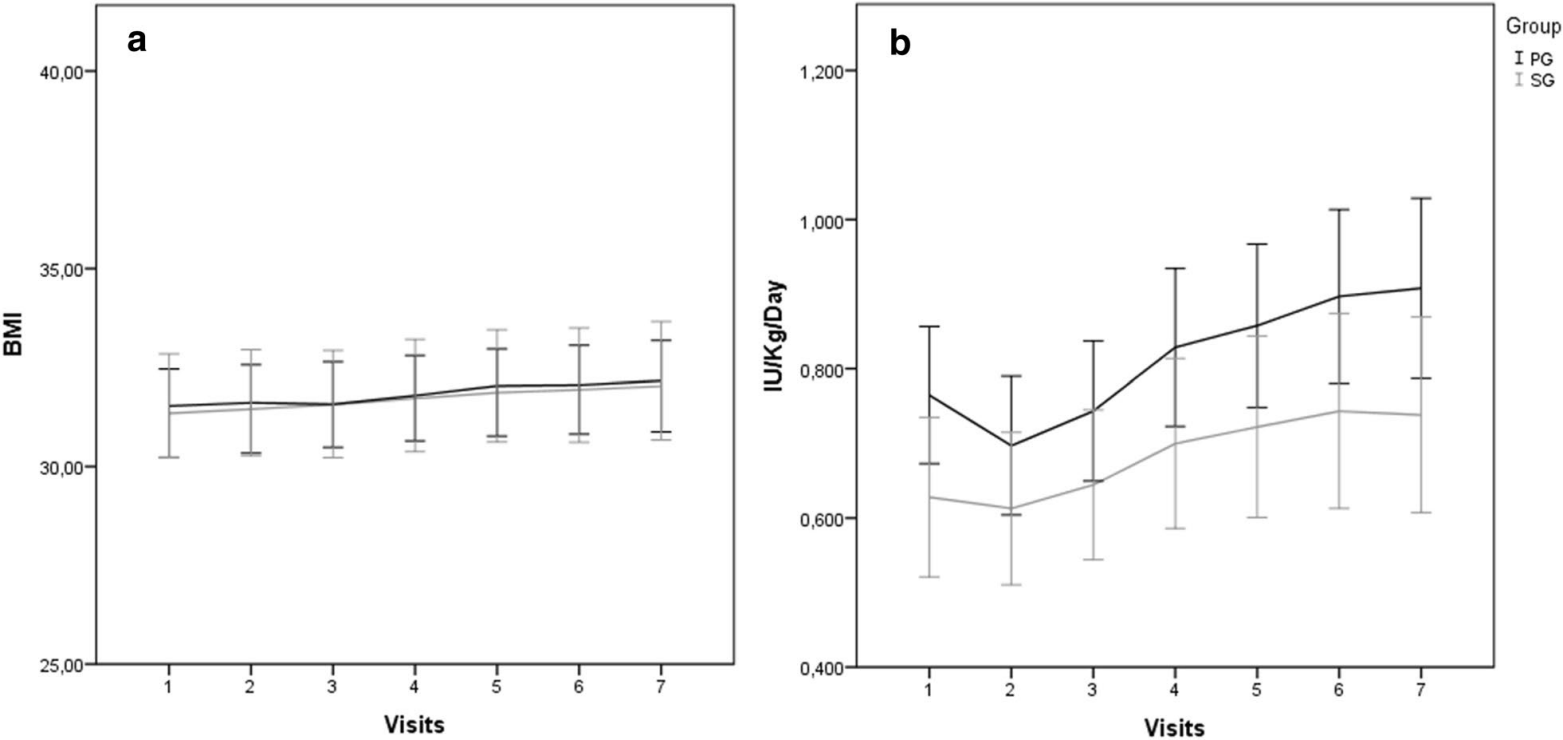
**A** Body mass index (BMI) *Mean* ± *SD*. **B** Quantity of used insulin per kilo per day. *Mean* ± *SD*

In relation to the prescribed dose of insulin, there was an increase in both groups during the study. Although more pronounced in PG, no difference was detected between the groups. PG used 0.76 ± 0.33 IU/kg and SG used 0.63 ± 0.39 IU/kg at baseline (p = 0.056). After 24 weeks, mean PG was 0.91 ± 0.47 IU/kg and SG was 0.74 ± 0.51 IU/kg (p = 0.059). There was no significant increase in weight over the 24 weeks. BMI was 31.35 ± 4.35 kg/m^2^ and 31.53 ± 5.03 kg/m^2^ (p = 0.059) at baseline. In 24 weeks, BMI was 32.03 ± 4.47 kg/m^2^ and 32.17 ± 5.54 kg/m^2^ (p = 0.898) in PG and SG, respectively. Details were described in Fig. 3.

We also examined the use of medications other than insulin. There was no difference between the number of drug classes throughout the study. We also found no difference in the number of pills ingested per day between the groups. However, at the first visit, PG used 1.17 ± 0.49 and SG 1.47 ± 0.62 (p = 0.004) classes of oral antihyperglycemic agents (in general, majority of patients used metformin and/or glibenclamide. These are the medications distributed for free in Brazil). At the end of the study, PG used 1.20 ± 0.55 and SG used 1.38 ± 0.61 (p = 0.086). In relation to classes of antihypertensives, PG used 2.80 ± 1.54 and SG used 2.58 ± 1.32 (p = 0.409) at baseline. After 24 weeks, PG used 2.97 ± 1.67 and SG used 2.60 ± 1.36 (p = 0.189). In Brazil, there are several antihypertensive drugs available for free. We do not perform class analysis. There were no significant differences in diastolic and systolic blood pressure values between groups during follow-up.

There was no difference in relation the number of patients using basal-bolus insulin scheme between the groups. At baseline, 27.87% and 31.67% were using basal-bolus insulin scheme in PG and SG, respectively (p = 0.694). At 24 weeks, the proportion was 42.62% in PG and 36.67% in SG (p = 0.461).

We evaluated Quality of Life through questionnaires [10, 11] applied on the first and last visits. There are no cut-off points defined in relation to patient performance. In this way, we compared the groups in these two moments of evaluation. PG had 37.62 ± 27.3 and SG had 28.46 ± 21.8 points at baseline (p = 0.044) and 25.90 ± 26.3 to PG and 25.31 ± 24.03 points to SG at last visit (p = 0.898). The higher score is worse in the influence of Diabetes on Quality of Life. Despite the difference found in the first visit, it does not appear during the study. To analyze the impact of disease and satisfaction with the treatment, we used the part of questionnaire DQOL. Other validated variables do not apply for elderly people. There was no difference between the groups and the first with the last visit.

## Discussion

In this RCT, the use of pens to apply insulin among elderly patients with uncontrolled T2D seems to reduce further glycated hemoglobin compared to the use of syringes. However, there was no difference in the other outcomes analyzed.

Both group of treatment had significant improvement in glycemic control over 24 weeks. This result reinforces that insulin, regardless of device used, improves the control. The superiority of pens can be understood based on the difference between baseline and 24 weeks. At the end of the study glycated hemoglobin was not statistically different between groups (8.39 vs 8.85% for PG and SG respectively). These final values for glycemic control are far off the ideal target for these patients. However, considering the participants were elderly, with a high prevalence of chronic complications, strict glycemic control could bring more risks [5, 6] and additionally in this study we did not have a target to reach.

The occurrence of hypoglycemia cannot be neglected. With advancing age, the number of episodes seems to increase, regardless of glycemic control [12]. We did not detect a difference in relation to the occurrence of hypoglycemia. High levels of HbA1c along the follow-up may contributed to the low frequency of hypoglycemia in both groups. Another factor that limited this analysis was the way of measuring hypoglycemia. The episodes were computed if recorded by patients. Unrecorded episodes could not be confirmed. Due to the small number of episodes, the presence of nocturnal, asymptomatic and severe hypoglycemia, we could only assess if the patient reported or not during the last month. There was no possibility of quantifying the number of severe, asymptomatic and nocturnal hypoglycemia episodes.

In relation to the evaluation of adherence, we were careful to accurately measure the number of insulin units used in each visit. The method used ensures that we find the closest value to the amount of insulin used in the previous month. Therefore, the degree of adhesion found in our study seems precise. We considered adequate adherence if the patient used at least 80% of the prescribed insulin dose in the previous month. Unlike patients with recent insulin use [4], method of application seems did not have much influence among patients using insulin for several years. Although there is no difference between groups, there was a tendency to be more adherent during the course of the study in both groups and our results were similar to other studies [5, 7, 12–15]. Training to use insulin in addition to frequent adjustments of insulin doses, may have contributed to this result.In association with the reduction of HbA1c, there was an increase in the amount of insulin used per kilo. We realize that the more frequent follow-up (monthly medical visits) in search of the glycemic target contributed to this result. At each visit, participants performed capillary blood glucose tests. Based on these notes, the researcher adjusted the dose of insulin. The low number of hypoglycemic episodes enabled us to increase insulin doses almost every visit. In addition, the apparent increase in PG may have been influenced by the ease of use of the method. Even without the blinded evaluator, we used a pre-defined protocol in both groups for treatment adjustment. However, there was an increase of 0.17 IU/kg/day in PG and 0.11 IU/kg/day in SG. These values appear not to have clinical significance, but should be considered.

In the Brazilian Public Health System, patients depend on free medication. In general, only metformin and sulfonylureas are available as oral drugs. For this reason, insulin is introduced into the treatment prior to prescribing other oral third class medication. In selected cases, even with metformin alone, insulin prescription is required to achieve satisfactory glycemic control. Either way, ADA [2] recommends the use of NPH and Regular insulin in developing countries, when the financial condition does not favor other treatment. We chose not to include patients in the use of insulin analogues for this reason. This study allows us to assess the reality of patients with diabetes in our country.

Recently, the use of insulin pens has been approved in Public Health System, which may also aid in treatment. Regarding the measurement of capillary glycemia, there is no consensus in the literature on the recommendation of frequency or effect among patients with T2D using insulin [16–18]. However, it is expected that for the proper adjustment of doses, this strategy will be present. However, it is not available to all patients in Brazil. In addition, access to public health care is a limiting factor in the Brazilian reality. Patients have specialist appointments every 4 to 6 months, with less frequent treatment adjustments than optimal to achieve satisfactory glycemic levels.

The greatest difficulty in relation to the assessment of Quality of Life and the impact of the disease was the interpretation of the questionnaires. Some of the patients had no perception of poor disease control and, for that reason, they responded as being satisfied with their health conditions. In a previous study, although patients had inadequate glycemic control, there was no perception of obstacles to treatment or understanding of the disease. [9] On the other hand, this perception made scores low on the first visit, not allowing the detection of improvement in quality of life during the study. Informally, patients who used pens appeared more satisfied throughout the study, but this was not reflected in the questionnaires. One of the probable reasons for this difference not being found is due to the small number of participants. In future studies, this assessment may be better understood. Despite the difficulties encountered, these questionnaires are validated for use in Brazil. All participants were able to answer completely.

Our main limitation was not to have blindness of the evaluators. Despite the use of care protocol to minimize the influence of this lack, there may have been influence on patient care and insulin doses. We chose not to blind the evaluator to ensure that all patients were treated, face to face, by the same investigator. The blinding of the researcher who made the treatment adjustment could harm the patient's medical relationship. Another limitation was the difficulty that some patients had to perform Self-monitoring of Blood Glucose due to visual or cognitive problems. In some cases, treatment adjustment was delayed. Some patients also had difficulty using their pens at first. However, at all visits the application technique was reviewed in both groups. Independently, patients who were not able to self-administer insulin were excluded before the intervention started. Regarding randomization, the initial treatment regimen was not stratified. However, the groups were quite similar, mainly in relation to the use of insulin and classes of oral antihyperglycemic agents. Regarding the statistical analysis, both groups showed similar results in absolute values of HbA1c in each evaluation. The large SD of the sample represents great variability, even with normal distribution, and small sample, despite the statistical power achieved with the calculated sample size. If the sample were larger, the SD could be reduced and even with a statistical difference found between the groups. The greatest reduction in PG was only in the delta of HbA1c reduction.

Few patients had not used insulin prior to the study. Analyzes without these patients were like results presented. Another limitation was the need to use a fixed protocol to adjust insulin doses. This option was taken to minimize the effect of the lack of blinding by the evaluators. The progressive increase in insulin doses was similar among patients, regardless of whether the glycemia value was higher or closer to the target. Thus, perhaps patients with less control needed more time to achieve blood glucose levels close to those desired.

## Conclusion

Comparing the use of pens with syringes to apply insulin among elderly patients with type 2 DM, we found no difference regarding the frequency of hypoglycemia, the need for higher doses of insulin or other medications. There was also no impact on Quality of Life. In both groups there was a reduction in HbA1c values in 24 weeks of follow-up, however with an absolute greater reduction in the group that used pens.

## Abbreviations

BMI: Body mass index
BPAID: Problems areas in diabetes–Brazil
CNPq: Conselho Nacional de Pesquisa
DM: Diabetes mellitus
DQOL: Diabetes quality of life
FIPE: Fundo de Incentivo à pesquisa
HbA1c: Hemoglobina glicada
HPLC: High performance liquid cromatography
IU/kg: International units per kilograms
Kg/m^2^: Kilograms per square meter
MDRD: Modification of diet in renal disease
mg/dl: Milligrams per decilitre
NPH: Neutral protamine hagedorn
PG: Pen group
SD: Standard deviation
SG: Syringe group
T2D: Diabetes mellitus tipo 2
